# Patient safety, self-injection, and B12 deficiency: a UK cross-sectional survey

**DOI:** 10.3399/BJGP.2021.0711

**Published:** 2022-10-04

**Authors:** Natasha Tyler, Alexander Hodkinson, Naeem Ahlam, Sally Giles, Andrew Zhou, Maria Panagioti

**Affiliations:** NIHR Greater Manchester Patient Safety Translational Research Centre, University of Manchester, Manchester, and NIHR School for Primary Care Research, University of Manchester, Manchester.; NIHR Greater Manchester Patient Safety Translational Research Centre, University of Manchester, Manchester, and NIHR School for Primary Care Research, University of Manchester, Manchester.; University of Manchester, Manchester.; NIHR Greater Manchester Patient Safety Translational Research Centre, University of Manchester, Manchester.; School of Clinical Medicine, University of Cambridge, Cambridge.; NIHR Greater Manchester Patient Safety Translational Research Centre, University of Manchester, Manchester, and NIHR School for Primary Care Research, University of Manchester, Manchester.

**Keywords:** cross-sectional studies, general practice, nutrition, patient perspectives, patient safety

## Abstract

**Background:**

Individuals with vitamin B12 deficiency (including pernicious anaemia) often report being ‘let down’ or stigmatised by general practice systems and policy, and choose instead to self-medicate via injection; the association between this and perceptions of safe primary care in this group of people is unknown.

**Aim:**

To examine the association between self-medication for vitamin B12 deficiency and patient-reported safety in primary care.

**Design and setting:**

A UK cross-sectional online survey.

**Method:**

The survey consisted of the three components: demographics; the validated Primary Care Patient Measure of Safety; and questions about self-medication for vitamin B12 deficiency. Multivariable logistic regression analyses and thematic synthesis were undertaken.

**Results:**

Responses from 1297 participants indicated 508 (39.2%) self-medicated via injection. Perceived primary care safety was low. Those who self-medicated via injection reported a significantly lower level of patient safety in primary care including adverse patient-related factors (odds ratio 0.82, 95% confidence interval = 0.73 to 0.92), and patients >34 years of age were significantly more likely to self-medicate via injection. Many reported that treatment under the guidance of a clinician was preferable to self-medication, but felt they had no other choice to regain quality of life. Almost half felt that the doctor did not always consider what they wanted for their care.

**Conclusion:**

To the authors’ knowledge, this is the largest study to date examining patient safety and vitamin B12 deficiency. It found that four out of 10 patients with B12 deficiency self-medicate via injection. Patients who self-medicated perceived primary care as less safe. Providing patient-centred care and treating these patients with dignity and respect is a policy priority to reduce unsafe health behaviours.

## INTRODUCTION

Vitamin B12 deficiency is a hidden condition that affects the quality of life of many people in the UK.^1^ It has an estimated prevalence of roughly 6% in the UK.^2^ People with vitamin B12 deficiency are primarily managed in primary care. Guidelines for diagnosis and treatment of vitamin B12 deficiency are inconsistent and many people are unable to access treatment.^3^ The most common cause of vitamin B12 deficiency is pernicious anaemia, an autoimmune disorder that results in inflammation and damage to the stomach lining.^4^

The deficiency is usually treated with injections of vitamin B12 in a form called hydroxocobalamin.^3^ Individuals must demonstrate deficiency in the diagnostic blood test to qualify for treatment but types of blood tests and ‘cut-off’ rates vary. Many patients describe their pressing need to access treatment as a continuous ‘battle’.^5^^,^^6^ As a result many people decide to self-medicate via injection (SMVI), without their GP’s knowledge or guidance and purchasing B12 ampules overseas without a prescription.^7^ Self-medication guided by GPs has numerous benefits such as reducing the primary care burden and improving patient empowerment. However, unguided self-medication has many potential risks such as incorrect self-diagnosis, dangerous drug interactions, incorrect manner of administration, severe adverse reactions, and masking of severe disease.^8^ An increasing number of GPs and patients advocate for changes to policy and practice that enables self-management of the disorder, including self-injection.^6^^,^^9^

Patient safety is a recognised concern in primary care^10^ especially for marginalised groups such as people with vitamin B12 deficiency.^11^ The most common contributory factors to safety in primary care are the quality of communication, diagnostics, and medication management.^10^ Patient-reported instruments have been developed and validated to identify the contributory factors to patient safety incidents in primary care.^12^^,^^13^ Individuals with vitamin B12 deficiency may be at greater risk of patient safety incidents in primary care as they often describe suboptimal communication, lack of dignity and respect, and feelings that healthcare professionals lack the knowledge, skills, and attitudes to adequately treat their condition.^5^^,^^12^^,^^14^^,^^15^

This study set out to:

- examine the association between patient-reported safety in primary care (with a focus on quality of communication and dignity) and self-medication in people with vitamin B12 deficiency controlling for clinical factors and sociodemographic factors;
- explore patient experiences surrounding self-medication in people with vitamin B12 deficiency and treatment changes following the COVID-19 pandemic.

**Table:** How this fits in

It is known that individuals with vitamin B12 deficiency (including pernicious anaemia) describe their primary care consultations as ‘battles’ and feel stigmatised. However, the extent of this dissatisfaction with primary care and the effect this might have on patient safety and unsafe health behaviours is unknown. This is the first study to assess the association between patient-reported safety and self-medication via injection and to consider the contributory factors to patient safety that affect this patient group. Understanding any negative effects of current practice and how GPs and primary care clinicians can better meet the needs of this marginalised group is key to improving safety and care.

## METHOD

### Design

This cross-sectional study consisted of an online questionnaire, distributed by an individual-participant-generated (unique) link using ‘Select Survey’. Data were anonymously collected from 16 July 2020 to 21 July 2020. STROBE statement guidance for reporting on cross-sectional studies was adhered to in this study.^16^

### Participants

Participants were recruited using advertisements in social media support groups for individuals with vitamin B12 deficiency/pernicious anaemia (see Acknowledgements). The only inclusion criterion for participation was diagnosis of vitamin B12 deficiency/pernicious anaemia; this was an initial screener question. If a diagnosis was not declared, a potential participant could not continue; there were no restrictions on the length of diagnosis. Participants were recruited from England, Wales, Scotland, Northern Ireland, and the Republic of Ireland. It was decided a priori that the questionnaire would only remain open for 6 days; as this was exploratory there were no study size decisions made a priori.

### Measures

The questionnaire consisted of three sections: demographics; the Primary Care Patient Measure of Safety (PC PMOS); and self-education.

### Sociodemographic characteristics

Diagnosis (vitamin B12 deficiency or pernicious anaemia), age, sex, education level, employment status, country of residence (England, Wales, Scotland, Northern Ireland, Republic of Ireland), region of residence (nine English regions, according to the Office for National Statistics, Wales, Scotland, and Northern Ireland) were assessed using the questions in Supplementary Table S1.

A single-item literacy screener was included to evaluate health literacy^17^ (‘How often do you need to have someone help you when you read instructions, pamphlets, or other written material from your doctor or pharmacy?’) and there was a single-item measure of health status^18^ (‘How would you rate your overall health?’).

Diagnosis (vitamin B12 deficiency or pernicious anaemia) was treated as a binary response in analysis, as everybody in the pernicious anaemia group was also classed as having vitamin B12 deficiency, as pernicious anaemia is the most common cause of vitamin B12 deficiency.^4^

### Patient-reported safety in primary care

A validated 28-item questionnaire was used to assess patient-reported contributory factors to safety in primary care (PC PMOS).^12^^,^^13^ The measure has 10 domains: dignity; patient-related factors; task performance; communication; access; information flow; external policy context; organisation and care planning; referrals; and physical environment. Some items were reverse coded; a larger score on each subscale indicates higher patient-perceived safety. The measures demonstrated good discriminant validity between primary care practices (*F* = 2.64, degrees of freedom = 72, *P*<0.001) and good internal validity for the domains; Cronbach’s alpha for most scales was >0.70. The scale almost demonstrated convergent validity, with a positive association with a staff measure of patient safety.^19^

### Self-medication

The measure for the dependent variable SMVI was developed for the purpose of this study. The questions were inspired by questionnaires from other self-medication questionnaires^20^^,^^21^ and patient and public engagement.^15^ SMVI was assessed using two items.

The first item was: *‘In general apart from treatments prescribed by your doctor do you sometimes take medications on your own to treat your B12 deficiency?’* The response options were ‘Yes’ or ‘No’. Followed by *‘If yes, which of the following do you take? (in addition to anything prescribed)’* with four responses: ‘self-injection’, ‘oral medication in tablet form’, ‘oral medication in liquid form’, ‘other, please state’.

Individuals who answered yes to item one and ‘self-injection’ to item two were coded as 1; all other responses were coded as 0. In addition, six multiple choice questions were asked (Supplementary Table S1).

### Free-text questions

A free-text question was included to gain a deeper understanding of circumstances around self-medication, ‘Do you have any concerns about safety and self-medication?’ There were also three other free-text questions relating to the treatment type that they were receiving, treatment frequency, and the effects of the SARSCoV-2 pandemic on their treatment.

### Analysis

Descriptive statistics included the number of participants and percentages for all the participants who took part in the survey. The association between discrete variables with diagnosis (that is, vitamin B12 deficiency/pernicious anaemia diagnosis) or self-medication (yes/no) was assessed by cross-tabulation and statistically by Pearson’s χ^2^-test or Fisher’s exact test. Only two participants had a missing primary outcome, which was imputed using MICE.^22^ To estimate the strength of the association, univariable (binary) logistic regression models were used to calculate the odds ratio (OR). Then a multivariable regression analysis was undertaken using the relevant variables associated with self-medication at the a-level of *P*<0.10 from the univariable analysis. Confidence intervals (CIs) reported are likelihood based. All *P-*values were two-sided, and *P*<0.05 was regarded as significant in the final multivariable model.

The *stats* package in R was used for the regression analysis. A Bayesian generalised linear model was used to check the consistency of the regression results using the R package *rstanarm*. Specifically, Markov Chain Monte Carlo sampling with four chains of 2000 iterations was used. Uninformative priors were used for the parameter in all models. All analyses were performed using R version 4.0.5 (R Foundation for Statistical Computing). For the corresponding primary outcome (SMVI), any missing values were imputed using the R package ‘MICE: Multivariate Imputation by Chained Equations’^22^ following Rubin’s principle for imputation.^23^ The baseline covariates (age, sex, and ethnicity) were used to predict missing data.

Free-text responses were analysed using thematic synthesis that consisted of three stages: line-by-line coding of text, development of descriptive themes, and generation of analytical themes (as outlined in Thomas and Harden^24^).

## RESULTS

### Descriptive characteristics

**Figure 1.**
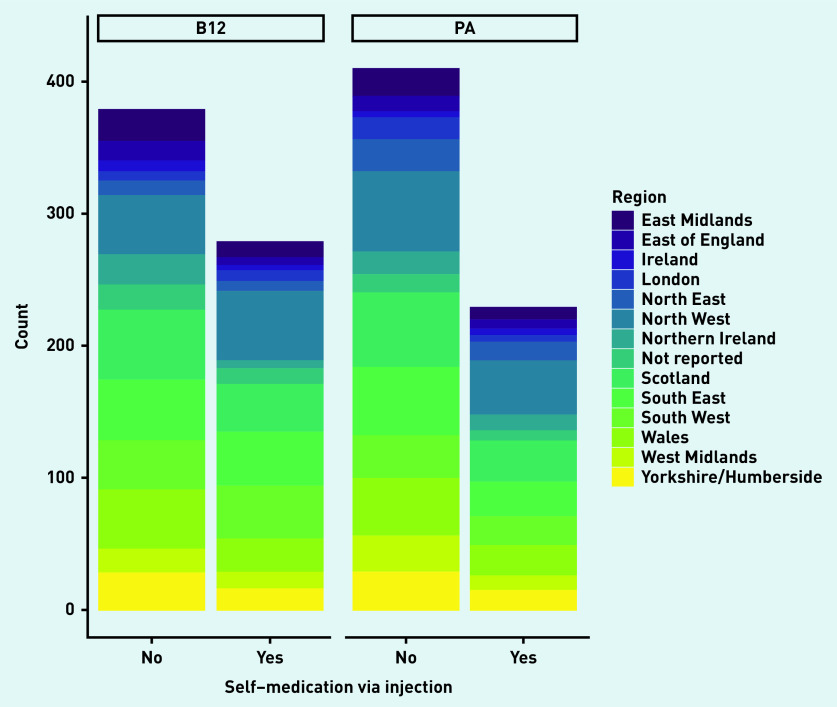
*Stacked bar chart to show medication via injection across regions. B12 = indicates B12 deficiency. PA = pernicious anaemia.*

The key characteristics of the 1297 responders are reported in Table 1. The sample was almost exclusively women (*n* = 1230, 94.8%) and over 82.9% of participants were aged ≥35 years (*n* = 1075). Approximately half of the participants had a diagnosis of pernicious anaemia (*n* = 639, 49.3%); the remainder had a vitamin B12 deficiency diagnosis only. Approximately three in 20 participants reported health literacy problems at least occasionally (that is, sometimes to always). Almost half of the participants were college or university educated (*n* = 568/1297, 43.8%) and 65.2% were employed (*n* = 846/1297). The areas of the UK and Ireland with the biggest representation were North West (197/1297, 15.2%), South East (165/1297, 12.7%), and Wales (137/1297, 10.6%) (Figure 1).

**Table 1.** General demographics of survey participants (*n* = 1297)

**Variable**	**Number (%)**
**Age, years (*n*= 1297)**	
18–34	222 (17.1)
35–44	337 (25.9)
45–54	426 (32.8)
55–64	224 (17.3)
>65	88 (6.8)

**Women**	1230 (94.8)

**Education level**	
College or university	568 (43.8)
Higher or secondary or further education	260 (20.0)
Postgraduate	233 (18.0)
Prefer not to say	44 (3.4)
Primary and secondary school	192 (14.8)

**Employment status**	
Employee or self-employed	846 (65.2)
Other	451 (34.8)

**Region^a^**	
East Midlands	67 (5.2)
East of England	39 (3.0)
Ireland	21 (1.6)
London	37 (2.9)
North East	57 (4.4)
North West	197 (15.2)
Northern Ireland	17 (1.3)
Scotland	176 (13.6)
South East	165 (12.7)
South West	130 (10.0)
Wales	137 (10.6)
West Midlands	69 (5.3)
Yorkshire/Humberside	88 (6.8)

**Diagnosis of vitamin B12 deficiency**	879 (67.7)

**Diagnosis of pernicious anaemia**	639 (49.3)

**Require support to read instructions, pamphlets, or other written material**	
Always/often	44 (3.4)
Sometimes	134 (10.3)
Rarely	134 (10.3)
Never	985 (75.9)

a *Some data are not reported or are missing.*

The characteristics of the sample did not significantly differ between those with a diagnosis of vitamin B12 deficiency compared with pernicious anaemia (Table 2). However, the association between self-medication and diagnosis type was significant (*P* = 0.002). There was also a significant difference between self-medication via injection and diagnosis (*P* = 0.016). There was no difference in total PC PMOS scores for the two diagnosis groups (vitamin B12 deficiency and pernicious anaemia; see Table 2).

**Table 2.** Characteristics of people with B12 deficiency including self-medication status/safety, type of diagnosis (B12 versus pernicious anaemia), general health status, and health literacy levels

**Variable**	**Diagnosis, *n* (%)**		χ**^2^-test (*P*-value)^a^**	**Paired *t*-test^b^ (*P*-value)**

	**Vitamin B12 deficiency**	**Pernicious anaemia**		
**Health status**				
Good/excellent	207 (16.0)	181 (14.0)	1.518 (0.218)	—
Fair/poor	451 (34.7)	458 (35.3)	—	—

**Health literacy (self-assessed)**				
Always/often	24 (1.9)	20 (1.5)	1.761 (0.624)	—
Sometimes	63 (4.9)	71 (5.5)	—	—
Rarely	73 (5.6)	61 (4.7)	—	—
Never	498 (38.3)	487 (37.5)	—	—

**Country of residence (region)**				
East Midlands	36 (3.5)	31 (3)	15.839 (0.147)	—
Ireland	12 (0.9)	9 (0.7)	—	—
London	15 (1.4)	22 (2.1)	—	—
North East	19 (1.8)	38 (3.7)	—	—
North West	96 (9.2)	101 (9.7)	—	—
Northern Ireland	17 (1.3)	7 (0.7)	—	—
Scotland	16 (1.5)	25 (2.4)	—	—
South East	87 (8.4)	78 (7.5)	—	—
South West	76 (7.3)	54 (5.2)	—	—
Wales	137 (10.6)	66 (6.3)	—	—
West Midlands	31 (3)	38 (3.7)	—	—
Yorkshire/Humberside	44 (4.2)	44 (4.2)	—	—

**Self-medication**				
Yes	435 (33.5)	368 (28.3)	9.997 (**0.002** )	—
No	222 (17.1)	270 (20.8)	—	—

**Self-medication via injection**				
Yes	279 (21.5)	229 (17.7)	5.862 (**0.016** )	—
No	379 (29.2)	410 (31.6)	—	—

**PC PMOS total**	—	—	—	−17.42 (0.071)

a *Pearson’s χ^2^ test for all factors. Bold text denotes significance at the 0.05 level.*

b *Paired* t*-test with 1298 degrees of freedom. PC PMOS = Primary Care Patient Measure of Safety.*

As many as 803 (61.9%) of the 1297 responders self-medicated with the majority via injection (*n* = 508/1297, 39.2%). Of the 803 who self-medicated, 63.3% (*n* = 508/803) self-injected and a few medicated using various oral methods. The most common reason for self-medication was to improve quality of life (*n* = 644/803, 80.1%), followed by dissatisfaction with treatment frequency (*n* = 545/803, 67.8%). Other reasons included concerns about overreliance on tests (*n* = 429/803, 53.4%) and lack of trust in healthcare professionals (*n* = 366/803, 45.6%). Participants could choose more than one option if they wished.

The most common source of information was an online closed support group (*n* = 577/805, 71.7%). Few participants who self-medicated informed a healthcare professional (437/803, 54.4%) did not. No participants reported side effects and the main symptoms participants aimed to improve were fatigue (*n* = 762/803, 94.9%), concentration/brain fog (*n* = 697, 86.8%) and pins and needles (*n* = 629/803, 78.3%); see Supplementary Table S2.

In terms of patient-reported safety in primary care, the participants in the current study had poorer perceptions of safety than the sample used in the PC PMOS validation study.^12^ This is indicated by the mean total PC PMOS scores and the mean scores of the individual domains (Supplementary Table S3).

**Figure 2.**
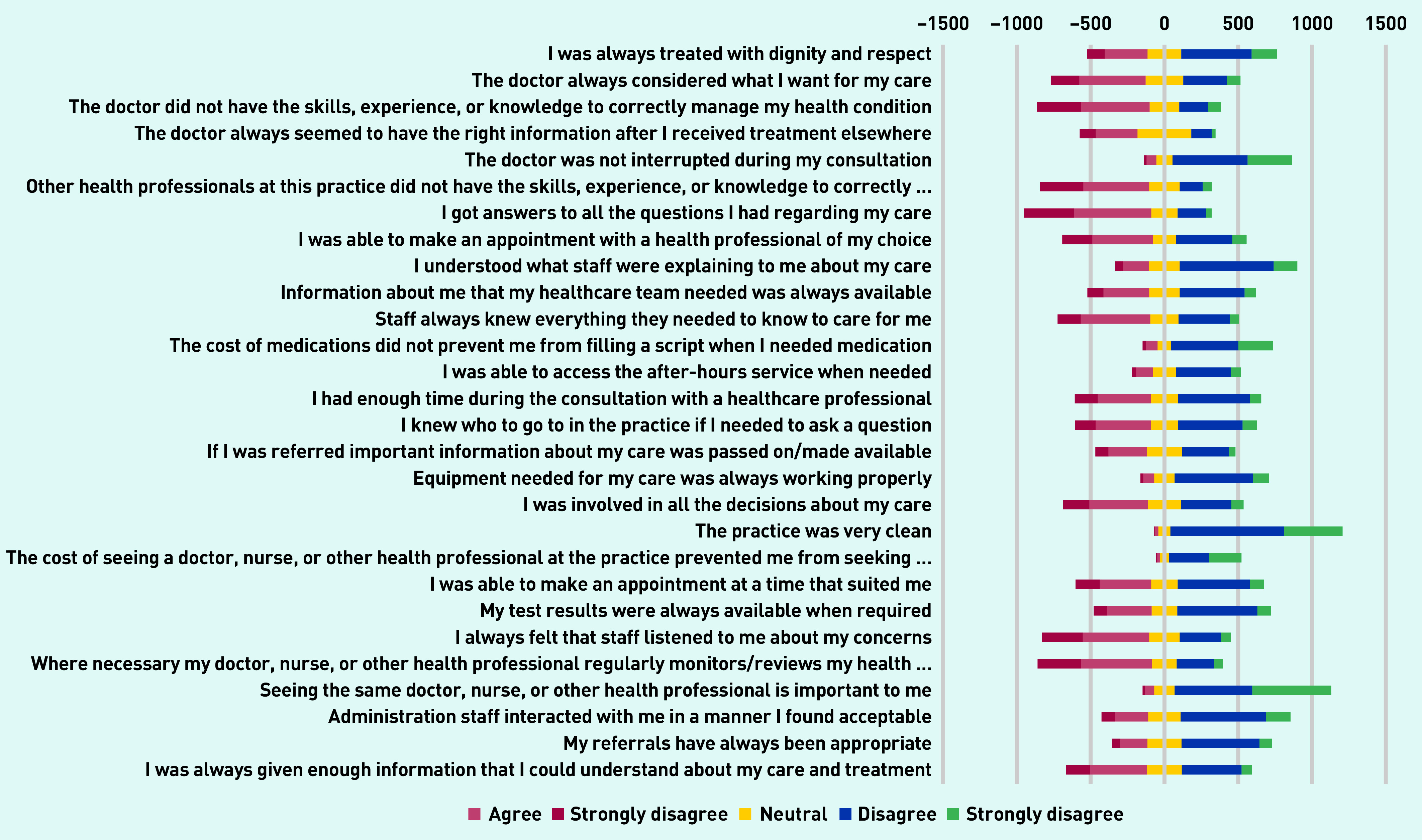
*Likert plot of PC PMOS responses. PC PMOS = Primary Care Patient Measure of Safety.*

Participants reported numerous safety concerns (Figure 2 and Supplementary Table S4). For example, only 50.0% (649/1297) of participants agreed that they were always treated with dignity/respect, 49.3% (640/1297) disagreed that the doctor always considered what they wanted for their care, 44.2% (573/1297) did not feel involved in decisions, 55.9% (725/1297) did not feel listened to, 42.3% (549/1297) felt they did not receive enough information, and only 18% felt they got answers to all questions about their care. Only 17.8% (231/1297) felt that staff knew everything they needed to care for them.

### Association between perceived primary care safety and self-medication by injection

The univariable logistic regression analysis showed that a lower total PC PMOS score (OR 0.97, 95% CI = 0.97 to 0.98) and lower scores on the individual domains (indicating lower perceived patient safety) were significantly associated with higher odds for SMVI (Supplementary Table S5). Other variables significantly associated with increased odds for SMVI included lower (poor/fair) health status (OR 1.46, 95% CI = 1.14 to 1.88), older age (≥45 years), and a pernicious anaemia diagnosis (OR 0.76, 95% CI = 0.61 to 0.95).

In multivariable regression analyses (Figure 2), two PC PMOS domains including patient-related factors (OR 0.82, 95% CI = 0.73 to 0.92), information flow (OR 1.10, 95% CI = 1.01 to 1.21), and external policy context (OR 1.10, 95% CI = 1.01 to 1.19) remained significantly associated with SMVI (Supplementary Table S6). All ages >34 years remained significantly associated with self-medication (age groups: 35–44 years OR 1.49, 95% CI = 1.01 to 2.20; 45–54 years, OR 2.06, 95% CI = 1.42 to 3.02; 55–64 years, OR 2.31, 95% CI = 1.51 to 3.55; ≥65 years, OR 2.80, 95% CI = 1.61 to 4.91).

**Figure 3.**
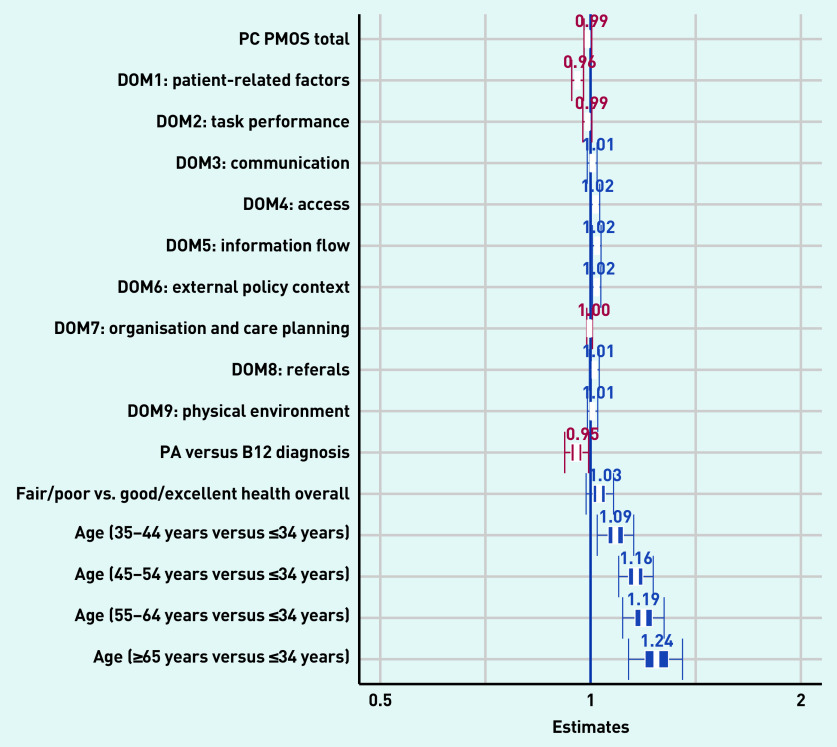
*Multivariate regression plot of the factors associated with self-medication by injection (Bayesian inference model). DOM = domain. PA = pernicious anaemia. PC PMOS = Primary Care Patient Measure of Safety.*

Variance inflation factor estimates indicated that total PC PMOS was 4.16, which indicates that this variable is moderately correlated with other variables in the model. The regression results that were checked through Bayesian inference showed very similar results (Figure 3).

### Thematic synthesis of patient concerns about self-medication and treatment implications of the COVID-19 pandemic

In total, 638 respondents completed the free-text question about self-medication concerns (see Supplementary Table S7 for a summary of the thematic synthesis). In total, 386 (60.5%) participants were not concerned about self-medication. The key thematic reasons for this were:

- experience;
- lack of trust in GPs;
- improved quality of life;
- adherence to guidelines; and
- credibility of sources.

The concerned group (192, 30.1%) had five key concerns:

- preference of health professional administration;
- no other choice;
- complications;
- storage and disposal; and
- financial concerns.

The remaining 60 (9.4%) responses were classified as indifferent or concerned (this group was largely categorised by patients who were initially concerned but were no longer concerned). The indifferent group presented three key themes:

- overcoming initial fears;
- social support; and
- type of injection.

Over half of the participants felt that the COVID-19 pandemic affected their care (749/1235, 60.6% [51 participants provided a ‘not applicable’ response, primarily because they no longer sought primary care provision because of dissatisfaction]). There were seven key themes surrounding those not affected:

- self-medication;
- proactive GP;
- proactive patient;
- receiving treatment in car;
- alternative treatment sufficient;
- direct contact with nurse practitioner; and
- location.

For those affected there were six key themes:

- appointment difficulties;
- treatment stopped or cancelled;
- monitoring and diagnosis stopped;
- alternative treatments not available;
- delayed/reduced frequency of injections; and
- effect on daily activities.

## DISCUSSION

### Summary

The current study found that four in 10 people with vitamin B12 deficiency/pernicious anaemia SMVI and they have lower than average perceptions of primary care safety. Those who SMVI perceived primary care as less safe and specifically they did not feel treated with dignity/respect or involved in decisions. Middle-aged and older participants were more likely to SMVI compared with younger participants.

The two most common reasons why participants chose to SMVI were to improve quality of life (80.0%) and because of dissatisfaction with current treatment frequency (67.8%). This research also highlights that the main source of information about self-medication is online closed support groups (71.7%).

Many of the participants did not want to SMVI and would prefer to be medicated under medical guidance, but felt they had no choice, as they wanted to regain quality of life. In line with similar research,^10^ six in 10 people experienced difficulties receiving their essential treatment during the pandemic.

### Strengths and limitations

To the authors’ knowledge, this is the first large-scale UK survey to assess patient safety in primary care for this patient group. However, participants were recruited from social media groups that 1) may not be representative of the older population who have a greater vitamin B12 deficiency prevalence and may use primary care frequently and 2) be more exposed to discussions around self-medication.

The cross-sectional nature of this study means that no causal inferences can be made about the direction of the associations. Furthermore, this study was conducted during the initial stages of the SARS-CoV-2 pandemic; many general practices stopped treatment.^9^ However, qualitative results (responses to free-text items in this study) show that SMVI was common before

This study relies on self-reported diagnosis and recruitment from online support groups that evidence suggests has some limitations. Groups sometimes have a high proportion of individuals who are struggling to gain a clinical diagnosis within primary care.^25^ Self-reported diagnosis could be representative of patients with subclinical cases, misdiagnosed individuals, or individuals who incorrectly attribute their symptoms.^25^ Research also suggests individuals in online communities may influence one another and potentially reaffirm negative views of healthcare systems.^26^

### Comparison with existing literature

To the authors’ knowledge, this is the first large survey examining perceived quality and safety in primary care for people with vitamin B12 deficiency/pernicious anaemia. The findings show that SMVI is an important safety concern often associated with suboptimal communication, feeling undignified in care interactions, and perceived lack of clinician knowledge or trustworthiness. The presence of these contributory factors is consistent with other research whereby patients described ‘battles’ and stigmatisation from practitioners.^5^

Patient safety for marginalised groups in primary care is a growing empirical field.^12^ Incident reporting systems are criticised because of underreporting of patient safety incidents and a tendency to focus on the proximal causes of incidents.^27^^–^^30^ The current research highlights the importance of patient-reported safety in primary care because they highlight issues that clinicians may not recognise^31^ and provide a framework for future directed learning to reduce safety incidents.^14^^,^^32^

### Implication for research and practice

Estimates suggests one in 20 people <60 years in the UK have vitamin B12 deficiency; this equates to roughly 3.4 million people. However, 20.0% of ≥60s could be affected.^2^ This is a marginalised group in terms of accessing care and the findings of the current study indicate that four out of 10 choose to SMVI, which unguided is an unsafe practice.

The current findings also suggest that self-injection might be driven by poor perception of primary care safety and especially low-perceived dignity and respect. The National Institute for Health and Care Excellence guidelines for pernicious anaemia are due to be published in 2023. There is a major need to develop/improve universal nationwide diagnosis and treatment policies for vitamin B12 deficiency in primary care. Such universal policies could reduce regional treatment discrepancies and help improve the relationships of these patients with their GP, and reduce unguided self-medication.

Finally, efforts to reduce the perceived stigma^6^ associated with this condition and encouraging clinicians to practice patient-centred care, driven by symptom recognition as opposed to reliance on suboptimal testing, could also increase patient safety. Future research should aim to confirm these results with more rigorous sampling methods and assess the perspectives of primary care practitioners in response to these results.
